# Impact of full-field digital mammography on diagnostic work-up and surgical management of mammographic microcalcification

**DOI:** 10.1186/bcr3275

**Published:** 2012-11-09

**Authors:** SM Bundred, J Zhou, S Whiteside, J Morris, NJ Bundred

**Affiliations:** 1 University Hospital South Manchester, Manchester, UK; 2 University of Manchester, UK

## Introduction

Full-field digital mammography (FFDM) increases detection of benign and malignant calcified breast lesions. Accurate nonoperative diagnosis of malignant impalpable breast lesions minimises numbers of therapeutic surgical procedures. Correct diagnosis of malignant mammographic microcalcification (MM) is important because upgraded lesions require repeat surgical procedures in 57% of cases (NHSBSP Audit 2012).

## Methods

Screening and symptomatic women with MM (*n *= 1,479) were reviewed to determine the impact of FFDM (imaging with FFDM only since April 2010) on the positive predictive value (PPV), diagnostic accuracy and surgical management of MM. Demographic information, preoperative and postoperative diagnosis and number of surgical procedures were recorded for Group 1 (August 2007 to March 2010: *n *= 711) and Group 2 (April 2010 to May 2011: *n *= 768).

## Results

Reduction in PPV of biopsy for MM was observed (Group 1, 42.6%: Group 2, 32.7%; *P *< 0.0001). Correct or concordant nonoperative diagnosis increased with FFDM (Group 1, 89% vs. Group 2, 95%; *P *< 0.0001) and was achieved more often at first attempt (Group 1, 80.6% vs. Group 2, 89.5%; *P *< 0.0001). More lesions under 5 mm were biopsied using FFDM (Group 1, 15%; Group 2, 20.4%; *P *= 0.008). Accurate preoperative diagnosis of malignancy permitted single-stage surgery in 77.4% Group 2 versus 67.9% Group 1 (*P *= 0.017). For DCIS cases, similar first-line mastectomy rates were observed (Group 1, 30.3% vs. Group 2, 33.3%, *P *= NS). Fewer B3/4 lesions upgraded at surgery (Group 1, 48.7% vs. Group 2, 20%; *P *= 0.011).

## Conclusion

Nonoperative work-up of MM using FFDM reduced second therapeutic procedures for MM, decreased upgrade of B3/4 lesions at diagnostic surgery, but increased benign nonoperative biopsies for MM.

